# Epigenetic Effects in HPA Axis Genes Associated with Cortical Thickness, ERP Components and SUD Outcome

**DOI:** 10.3390/bs12100347

**Published:** 2022-09-20

**Authors:** Shirley Y. Hill, Jeannette L. Wellman, Nicholas Zezza, Stuart R. Steinhauer, Vinod Sharma, Brian Holmes

**Affiliations:** 1 Department of Psychiatry, University of Pittsburgh School of Medicine, 3811 O’Hara St., Pittsburgh, PA 15213, USA; 2 Department of Psychiatry and Magee Women’s Hospital, University of Pittsburgh Medical Center, Pittsburgh, PA 15213, USA; 3 Department of Psychiatry and Shadyside Hospital, University of Pittsburgh Medical Center, Pittsburgh, PA 15213, USA; 4 Pittsburgh VA Medical Center, University Drive C, Pittsburgh, PA 15240, USA; 5 UPMC Children’s Hospital of Pittsburgh, 4401 Penn Ave., Pittsburgh, PA 15224, USA

**Keywords:** alcohol use disorder (AUD), event-related potential (ERP), early life adversity (ELA), methylation, epigenetic, CRHR1, stress, cortical thickness

## Abstract

Association between familial loading for alcohol use disorders (AUD) and event-related potentials (ERPs) suggests a genetic basis for these oscillations though much less is known about epigenetic pathways influenced by environmental variation. Early life adversity (ELA) influences negative outcomes much later in life. The stress-activated neuropeptide corticotropin-releasing hormone (CRH) contributes to the deleterious effects of ELA on brain structure and function in animals. Accordingly, we hypothesized that ELA would be related to cortical thickness and electrophysiological characteristics through an epigenetic effect on CRH receptor type-1 (CRHR1) methylation. A total of 217 adolescent and young adult participants from either multiplex alcohol dependence or control families were scanned using magnetic resonance imaging (MRI) at 3T and cortical thickness was determined. Longitudinal follow-up across childhood, adolescence, and young adulthood provided developmental ERP data and measures of adversity. Blood samples for genetic and epigenetic analyses were obtained in childhood. Cortical thickness and visual ERP components were analyzed for their association and tested for familial risk group differences. Visual P300 amplitude at Pz and cortical thickness of the left lateral orbitofrontal region (LOFC), were significantly related to risk group status. LOFC cortical thickness showed a negative correlation with CRHR1 methylation status and with childhood total stress scores from the Life Stressors and Social Resources Inventory (LISRES). Stress scores were also significantly related to P300 amplitude recorded in childhood. The present results suggest that early life adversity reflected in greater total LISRES stress scores in childhood can impact the methylation of the CRHR1 gene with implications for brain development as seen in cortical thickness and electrophysiological signals emanating from particular brain regions.

## 1. Introduction

Alcohol use disorder (AUD) is a chronic relapsing condition with significant health consequences. Alcohol use has been reported to be the seventh leading risk factor for death and disability globally and the primary factor in mortality for those 15–49 worldwide [1,2]. There has been a long-standing interest in identifying biomarkers of risk for AUD to provide opportunities for identifying those at greater risk for developing AUD and related substance use disorders. 

Electroencephalography (EEG) has a long history in neurology and psychiatry because it can provide an indication of neurobiological alterations associated with dysregulation and/or disease states. The EEG can reflect sensory processing effects but also more complex aspects of cognitive processing. The event-related potential (ERP) is a wave that is time-locked to the onset of a specific stimulus that reflects both registration that the stimulus has arrived at the cortex reflected in the early components (N1, P1), but also the late components including P300 which indexes more complex processing. The ERP was first identified in 1965 by Sutton and colleagues [3]. Since that time, a number of components of the ERP wave have been identified and studied in relation to cognitive processing characteristics of individuals with various psychiatric syndromes including schizophrenia, depression, and alcohol use disorders [4,5,6]. Those at familial genetic risk for these disorders have also shown ERP wave variation, particularly in the P300 component starting with the seminal report by Begleiter and colleagues in 1984 [7] and replicated in many other laboratories including ours [8]. Continued research of ERP and P300 in particular has shown that externalizing behavioral traits in parents may be the salient aspect of parental alcohol use disorder that is associated with P300 variation in offspring [9]. Psychopathology in offspring is also associated with a reduction in the amplitude of the P300 component [10]. Longitudinal assessment of ERP and substance use disorder outcome has now shown that P300 amplitude in childhood may have an important predictive value for determining SUD status by young adulthood [11,12]. 

The P300 component of the ERP is a scalp positive wave that occurs approximately 300 ms after an informative stimulus occurs. Typically, a participant is instructed to attend to one stimulus (target) while ignoring another as completed in “oddball” paradigms. Stimulus relevance, and the frequency and probability that the target stimulus will occur, influence the recorded amplitude of the P300 component. P300 was first thought to be an index of capacity to attend to and process stimulus information [13,14]. More recently it has been demonstrated that P300 amplitude is affected by the stimulus paradigms presented, the age and gender of participants, and a host of other variables including genetic factors [15,16,17,18].

Because recent studies have focused on EEG oscillations, particularly delta and theta oscillations [19,20], comparisons with P300 component studies are described here. P300 response is primarily the outcome of delta (1.0–3.0 Hz) and theta (3.5–7.5 Hz) oscillations during the cognitive processing of stimuli (see [21] for review). These oscillations in delta and theta are not state-dependent effects seen only in individuals with an AUD but rather can be assumed to be a familial trait because they are seen in high-risk relatives of individuals with AUD [22,23]. 

**Familial Risk for AUD and P300 Trajectories in Childhood:** Many early cross-sectional studies reported P300 amplitude reduction in children from families in which alcohol use disorders are present in the parent generation in comparison to offspring from control families [7,8,24,25]. With the expectation that P300 amplitude may change with age, one early study from our lab [26] modeled the trajectories of P300 amplitude using repeated ERP assessments (four or more times) in child and adolescent offspring, contrasting youngsters with multiple cases of AUD and those at low familial risk for alcohol use disorder (control families). The developmental trajectories of P300 in childhood that differed by familial risk group status in this study [26] were also related to the presence of child/adolescent psychiatric status. These studies suggested that while the amplitude of P300 at one point in time may reveal risk for later development of substance use disorders, age-related change in P300 amplitude may provide a more salient biological marker of AUD risk in youth than single point measures. 

Trajectory analyses have been completed in other studies [11,18,27] that have extended into young adulthood where P300 was collected at multiple time points and the outcome of participants was noted. In one study [27] in which data were collected at three points in time (ages 17, 20, and 23), P300 amplitude in offspring was found to decrease over time with age and with the severity of externalizing disorder in their parents. Using P300 at multiple points during childhood and adolescence revealed that P300 amplitude at age 10 is predictive of substance use disorder outcome at age 20 though P300 at later ages was not [11]. Trajectory modeling has confirmed previous reports of an association between the low visual P300 trajectory with high familial risk especially in male offspring [18] in the 8–12 age range, but not in the 13–18 or 19–29-year-old age range.

Although long-term longitudinal studies are in agreement that P300 amplitude is associated with familial risk for AUD, there is some variation in the emphasis that has been placed on whether a reduction in P300 amplitude is an endophenotype exclusively or predominantly representing an externalizing behavioral dimension that, in turn, increases the risk for a variety of antisocial behaviors [28,29,30,31,32] including alcohol use disorder and substance use disorder. 

P300 reduction may be an endophenotype of risk for developing AUD or other SUD because of an overall greater likelihood of experiencing child/adolescent psychopathology which, in turn, elevates the risk for subsequent AUD or substance use disorders. Childhood psychopathology whether externalizing or internalizing is associated with lower P300 amplitude [26] with any childhood psychopathology associated with a greater likelihood of being in the low amplitude trajectory class [10]. Interestingly, children with internalizing disorders had lower P300 amplitude, and those with both internalizing and externalizing disorders had the lowest P300 amplitude [26]. Similarly, Van Der Stelt [33] found both internalizing and externalizing disorders moderated the effect of family history on P300 amplitude.

The internalizing dimension has received less attention overall in studies of children and adolescents with familial risk for AUD, with most measuring the externalizing dimension exclusively [29,30,31,34]. The presence of a depressive disorder is associated with a reduction of P300 amplitude in adults [4,5,35,36,37] suggesting that childhood depression could influence the amplitude of the P300 component as seen in our longitudinal analysis [26].

Demonstration that P300 amplitude is heritable [27,38,39,40,41] has offered justification for the pursuit of genetic variation associated with ERP components and more broadly in studies of delta and theta brain oscillations. A number of studies have now found genetic variation associated with brain oscillations. Visual P300 has been associated with variation in the dopamine D2 receptor locus on chromosome 11 [42], SNPs within the CHRM2 gene on chromosome 7 [18,22,43], and GABRA2 variation on chromosome 4 [44].

**Genes, Environment, and P300:** Although the evidence for genetic variation impacting the production of brain oscillations is clear, less is known about the degree to which environmental factors can impact brain electrophysiological responses. **We speculate that** the association between P300 amplitude and childhood disorders may reflect both genetic and environmental susceptibility. Specifically, the home environment of children from families coping with addiction may present adversity that results in reported symptoms of childhood disorders which, in turn, may change the electrophysiological characteristics of those who experience these difficulties. The childhood environment has been shown to be associated with brain structural characteristics suggesting that electrophysiological changes due to environmental variation are a clear possibility.

**Childhood Adversity and Brain Structure:** Childhood maltreatment has been broadly defined to include physical or emotional abuse, sexual abuse, and neglect. Experiencing a variety of adverse environmental conditions has been associated with multiple negative mental and physical outcomes [45,46]. Structural brain abnormalities have been pursued to evaluate the impact of adverse conditions in childhood with most finding changes associated with abnormalities, particularly in cortical thickness [46,47,48,49,50,51]. Cortical surface area and thickness are related to cortical volume but each display separate developmental and genetic associations [52]. Increased cortical thinning has been reported in adolescents who are family history positive for alcohol use disorder [53]. The developmental goal during childhood and adolescence is to display cortical thinning so it is unclear if these findings have the same meaning as cortical thickness in young adults or adults where thinning will have reached a plateau unless adverse disease processes intervene [54]. 

**Figure 1:**
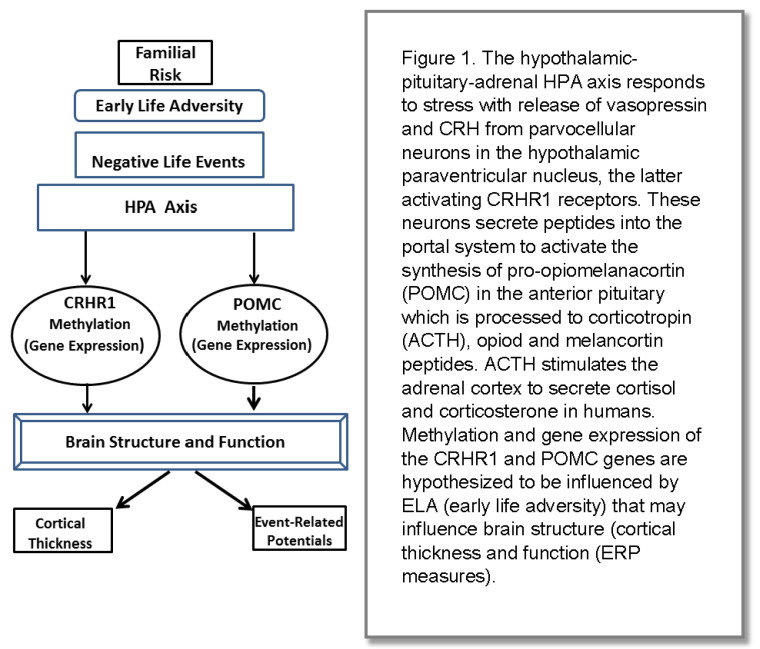


**Childhood Adversity, Neuroendocrine Mechanisms and Epigenetics:** We hypothesize that one aspect of having familial risk for developing AUD and related SUD is the experience of early childhood adversity. This adversity that creates a stress response may be translated into epigenetic changes in genes that in turn affect brain structure and functioning (Figure 1).

The stress reaction is mediated through the hypothalamic–pituitary–adrenal (HPA) system. The hypothalamus releases corticotrophin-releasing hormone (CRH) when an individual experiences stressful signals from the environment. The hormone binds to the corticotropin-releasing hormone receptor 1 (CRHR1) in the pituitary [55]. The proopiomelanocortin (POMC) gene produces two classes of peptides, melanocortin, and β endorphin, with functions ranging from energy homeostasis, immune system function, and stress response. When released from the hypothalamic paraventricular nucleus, CRH activates the synthesis of POMC in the anterior pituitary and is processed into several peptides including adrenocorticotropin hormone, triggering the release of cortisol from the adrenal cortex. Glucocorticoid receptors provide negative feedback loops binding cortisol to dampen HPA activity. Epigenetic mechanisms can alter the functioning of the HPA axis by changing gene expression. Among the mechanisms leading to epigenetic modification, DNA methylation has been studied the most extensively. In general, greater methylation leads to reduced gene expression whereas lesser methylation is associated with increased expression. The relationship between early life adversities and changes in components of the HPA axis has been studied extensively [46,47,48,49,50,51].

**Integrating Brain Structural Characteristics and P300:** Cognitive development from childhood to adulthood is accompanied by changes in electrophysiological characteristics with event-related potentials (ERPs) being among the most widely studied neural components [56]. Similarly, brain structural changes seen during development have frequently been studied [57,58,59,60]. The study of electrophysiological characteristics in the context of brain morphology has been far less frequent. Efforts to relate P300 to brain morphology have been reported in relation to aging effects [61,62,63] and developmental effects from childhood to adulthood [64].

The present set of analyses utilized data from a valuable cohort selected for longitudinal assessment of psychopathology **using semi-structured interviews [65] in childhood and adolescence**. The participants were members of families identified through a proband with multiple relatives with alcohol dependence (AD) (multiplex for alcohol dependence families) or an index case with an absence of AD members (control families). The offspring from the proband generation participated in longitudinal data collection spanning childhood, adolescence, and young adulthood. Because these offspring from multiplex families show an increased risk for developing substance use disorders relative to offspring of controls [66], it was of interest to determine the impact of familial background on brain morphology and functioning and relate these to childhood indicators of potential adversity. Prospective assessment of stressors and availability of resources for potentially ameliorating stressors provided data for determining whether environmental variation might influence brain morphology and electrophysiological response based on event-related potentials. 

## 2. Methods

### 2.1. Participants

A total of 217 adolescent and young adult participants from high (N = 113) and low risk (N = 104) alcohol dependence families were analyzed for the present report. The prospective longitudinal family study of third-generation offspring included multiple assessments at approximately yearly intervals in childhood and biennially in young adulthood. Families were selected through the parent generation and included collection of data for the 1st generation grandparents. Selection of multiplex families was based on the presence of two same-sex adult siblings with AD within the family. These multiplex families were considered to be at high risk for transmission of alcohol and other substance use disorders within the family. Control families were selected on the basis of having two same-sex adult siblings and minimal familial psychiatric disorders including substance use disorders within the family. The study has ongoing approval from the University of Pittsburgh Institutional Review Board. All participants provided consent at each visit. Children provided assent with parental consent. 

#### Follow-Up Samples

Children/adolescents between the ages of 8–18 years who were the offspring of second-generation family members were eligible to participate. Participants enrolled in the child/adolescent follow-up completed repeated assessments until age 19. With the majority of these continuing their participation in a young adult follow-up, neuroimaging assessment at an average of 25.3 ± 4.9 years was completed. Demographic characteristics of the subsamples are seen in Table 1.

### 2.2. Clinical Assessment

Clinical diagnoses obtained during childhood/adolescence (before age 19) were determined by administering the **Kiddie** Schedule for Affective Disorders and Schizophrenia (K-SADS), Ref. [65] followed by a best estimate consensus diagnosis [66]. To provide normative data for childhood assessment of psychopathology, the Child Behavior Checklist was also administered. **Participants remaining in the follow-up until their 19th birthday were assessed in a young adult protocol that included administration** of a structured psychiatric interview, the Composite International Diagnostic Interview (CIDI) [67] providing DSM-IV diagnoses including major depressive disorder. 

### 2.3. Childhood Adversity: Life Stressors and Social Resources Inventory (LISRES)

Administration of the Life Stressors and Social Resources Inventory (LISRES) [68] was completed at each follow-up visit during childhood. The instrument assesses stressors and social support from eight sources: physical health, home and neighborhood, parents, sibling, extended family, school, and boyfriend/girlfriend. A total score is calculated for stressors, the Negative Life Events scale, and a total score is determined for the same eight sources from which positive support was reported resulting in the Positive Life Events scale. These scores were included in the overall analysis due to the potential for familial alcohol dependence to be associated with the environmental milieu that might alter brain volumes based on the now large literature suggesting that early environment can have effects on brain structure [46,47,48,49,50,51].

### 2.4. Event-Related Potentials: Visual Task

Electrophysiological data were amplified by 20 k using a Grass Model 12 Neurodata system set to a bandpass of 0.01 Hz to 30 Hz. Each trial was sampled for 1200 msec at 8-msec intervals beginning with the 200-msec prestimulus baseline. Each child performed a visual ERP task with electrodes placed at frontal, vertex, parietal, and occipital locations (Fz, Cz, Pz, Oz, P3, P4). All the active electrodes were referred to as linked ears with a forehead ground. Eye blinks were tracked online using an oscilloscope and all trials affected by eye artifacts (blinks or eye movements greater than 50 mv) were excluded online. Only the artifact-free trials were averaged according to conditions offline. The visual task consisted of presentation of a brief (33 ms) target (stick figure head with a nose and only one ear oriented with nose upward or nose downward) or a nontarget (circle) stimulus. The subject responded to the position of the ear (left or right) with a button press (left or right). Targets occurred on 80 of the 240 trials presented. The task was originally used in the Begleiter Neurodynamics laboratory [7] and subsequently tested in multiple labs investigating the P300 ERP component, finding an association with familial risk for alcohol use disorders and externalizing psychopathology [69], and predicting substance use disorder by age 20 [11].

### 2.5. Epigenetic Data: DNA Isolation and Methylation Assays

Genomic DNA was utilized from a resource extracted from whole blood or from EBV transformation and cryopreservation. Using EpiTect Bisulfite kits, DNA was converted and cleaned. PCR amplification of the CRHR1 and POMC regions was completed using thermal cycling that followed Qiagen guidelines for these predesigned primers. (Hs_CRHR1_02_PM Pyromark CpG assay) (PM 00066136) and (Hs_POMC_01_PM PyroMark CpG assay) (PM 00007910).

CRHR1 Sequence Analyzed: CGTTCGCGTCTCAGCGCGC. The CRHR1 sequence is located on Chromosome 17. BP 438610XX-43 8610XX.

POMC Sequence Analyzed: GCGTTGGGTCTCCGCTTAGAACGGGCGGGA. The POMC sequence is located on Chromosome 2. BP 25,391,6XX-25,391,7XX. 

### 2.6. Imaging Parameters

The MRI scans were performed at the University of Pittsburgh Medical Center (UPMC) Magnetic Resonance Research Center (MRRC) using a 3T head-only Siemens Trio scanner (Siemens Medical Systems, Ehrlangen, Germany) equipped with a fast gradient system for echoplanar imaging. A standard radiofrequency head coil was used with foam padding to restrict head motion. A 7 min 3D T1-weighted Magnetization Prepared Rapid Gradient Echo Imaging (MPRAGE) sequence (TR = 2300 ms, TE = 2.98 ms, FA = 9 deg, field of view FOV = 240 mm, acquisition matrix = 240 × 256, in-plane resolution 1.0 × 1.0 mm^2^, yielding 160 transversal slices with a thickness of 1.2 mm) was used to acquire a high-resolution anatomical scan for FreeSurfer analysis. 

#### FreeSurfer Analysis

Cortical reconstruction and volumetric segmentation were performed using the recon_all option in the FreeSurfer image analysis suite. FreeSurfer (version 5.3, Fischl, B; Boston, USA) (https://surfer.nmr.mgh.harvard.edu) was used as previously described [70]. All scans were assessed for movement and noise artifacts. The automated processing included motion correction and averaging of multiple T1 weighted images [71], removal of non-brain tissue [72], automated Talairach transformation, and segmentation. Segmentation was directed at subcortical white matter and deep volumetric structures. Intensity normalization, tessellation of the gray matter/white matter boundary, automated topology correction, and surface deformation were performed by following the intensity gradients of the gray/white and gray/cerebrospinal fluid borders to optimally place them at the location of greatest shift in intensity for defining the transition to a new tissue class.

Following the development of the cortical models, data pre-processing also included surface inflation [73], registration to a spherical atlas, and parcellation of the cerebral cortex into units with respect to gyrus and sulcus structure [74,75,76] providing the creation of a variety of surface-based data (e.g., curvature and sulcal depth). In FreeSurfer, the Desikan–Killiany automated labeling system was used to parcellate the PFC [74]. This provides measures of volume, surface area and cortical thickness. The focus of our analysis was on cortical thickness. The procedures available in FreeSurfer for measuring cortical thickness have been validated against histological evidence [77] and manual tracing methods [78,79].

## 3. Results

### 3.1. Demographic Characteristics

Demographic characteristics were analyzed to determine if the familial risk groups differed significantly. The groups varied in age at the MRI scan, at the age when the nearest ERP assessment to the MRI was obtained, and at the age of the last follow-up, though all differences averaged only about two years (Table 1). The age of the first ERP assessment in childhood did not differ, however. Significant differences were seen for socioeconomic status (SES) and scores on the Peabody Picture Vocabulary Test (**PPVT**) with low-risk controls showing higher SES and PPVT scores (Table 1). Of relevance to the imaging data acquired, significant differences in the number of right-handed individuals by risk group were not seen. Additionally, the groups did not differ significantly in BMI. The presence of DSM-IV drug abuse or drug dependence differed significantly by familial risk with a greater proportion of high risk experiencing a lifetime diagnosis by the age of last follow-up (Table 1), a pattern also seen prior to the MRI scan.

### 3.2. Cortical Thickness in FreeSurfer Regions and ERP Components

Cortical thickness values for 34 regions in each hemisphere were correlated with ERP components obtained at the time point closest to the date of the MRI scan. From these two correlation matrices, correlation values <0.01 were tabulated. Those meeting this criterion were then tested for whether or not these variables differed by familial risk. Results of this culling process can be seen in Table 2 for the left hemisphere and in Table 3 for the right hemisphere.

Table 4 illustrates the familial risk group differences for these variables that supported the selection of variables shown in Table 2 and Table 3.

### 3.3. ERP and Cortical Thickness: Predictors of Substance Use Disorder

**Figure 2:**
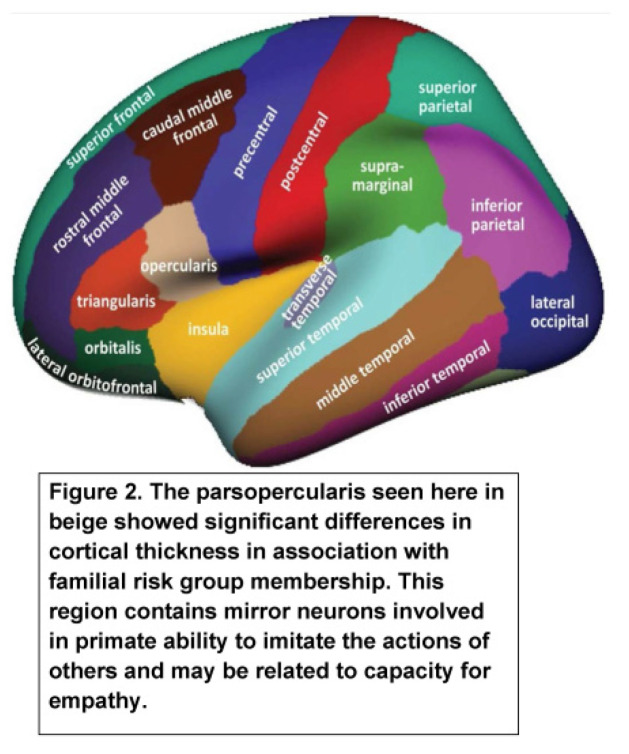


**Figure 3:**
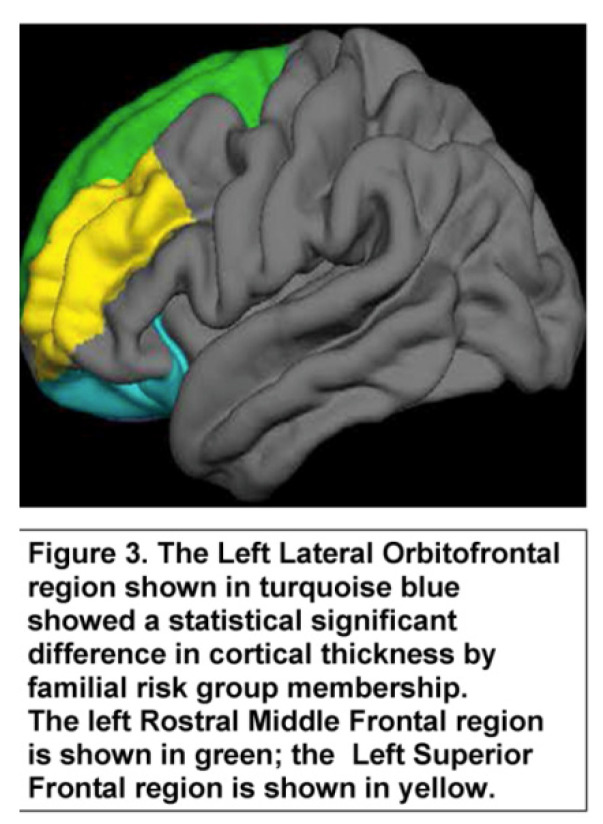


The goal of this analysis was to determine if ERP components at the earliest assessment, those acquired closest to the scan, and our two most salient FreeSurfer regions, Rh parsopercularis (Figure 2) and Lh lateral orbitofrontal (Figure 3) cortical thickness, would predict SUD. 

The parsopercularis (Figure 2) corresponds to Brodmann area BA44 in the dominant hemisphere. The primary function of this region is language production and phonological processing. This region is also noted for the presence of mirror neurons. The present results find significant correlations between the cortical thickness of this region with visual P300 amplitude.

One of the notable functions of the lateral orbitofrontal region (Figure 3) is to anticipate choices and integrate prior experience with current information. The cortical thickness of the lateral orbitofrontal region in the left hemisphere was significantly correlated with visual P300 amplitude at Pz within each of the risk groups though a somewhat higher correlation was seen in the low-risk group.

The analysis was performed using a Cox regression analysis and a Wald backward procedure applied. ERP variables collected at the first assessment and those closest to the scan were analyzed along with the thickness of the parsopercularis (right hemisphere) and thickness of the lateral orbitofrontal region in the left hemisphere as potential predictors of SUD. All variables but two (N2 amplitude at Cz earliest and Lh lateral orbitofrontal thickness) remained in the equation (Table 5). All of the remaining variables were significant although the thickness of the parsopercularis was only marginally significant.

### 3.4. Childhood Adversity, Methylation of the CRHR1 Gene, and Cortical Thickness

Administration of the LISRES in follow-up visits during childhood provided an opportunity to evaluate potential childhood adversity and its influence on epigenetic changes in the CRHR1 gene and its impact on the thickness of the regions showing significant familial risk group differences. The analysis focused on the Negative Life Events **(NLE)** score obtained either before the time of the DNA collection (N = 87) or either before or after the DNA collection (N = 176) but within a few years of the collection. Significant familial risk group differences were found for CRHR1 methylation, NLE scores proximal and before DNA collection, or NLE scores in close proximity to DNA collection (Table 6). Familial risk group differences were also seen in the thickness of the lateral orbitofrontal (Lh) and parsopercularis (Rh) regions (Table 6).

### 3.5. SUD Outcome, NLE, CRHR1 Methylation, and Cortical Thickness

Survival analysis for the age of onset of SUD and its associated survival time was first conducted as a “proof of concept” to investigate whether methylation of the CRHR1 gene was potentially influenced by the childhood experience of adversity, and more specifically as measured by negative life events obtained before the DNA sample was collected for methylation processing. CRHR1 methylation showed a Wald value of 16.98, df = 1, *p* < 0.001 with a beta of −5.88 and Exp (B) of 0.003. CRHR1 methylation as interaction with negative life events before collection of DNA provided a Wald value of 25.03, df = 1, *p* < 0.001 with a beta of 0.083 and an Exp (B) of 1.086. These results demonstrate that childhood stressful events resulted in an 8.6% change in SUD onset and SUD outcome.

With the demonstration that negative life events occurring before DNA sampling had an interactive effect on methylation, it was of interest to model this relationship in the context of the cortical thickness of primary regions of interest. Because the cortical thickness of the Rh parsopercularis and the Lh lateral orbital region were highly correlated (r = 0.42, df = 1, *p* < 0.001), separate models were tested each including either Lh lateral orbitofrontal or Rh parsopercularis. Cox regression models were used to evaluate the effects of negative life events and methylation of CRHR1 first with the parsopercularis (Rh) (Model 1), followed by analysis with the lateral orbitofrontal thickness (Lh) (Model 2). Model 1 results showed minimal effects of methylation alone or Rh parsopercularis cortical thickness on SUD outcome (Table 7). In contrast, the interaction of CRHR1 methylation and Rh parsopercularis showed a significant effect, beta = −8.31 (*p* = 0.056) with an associated Exp (B) <0.001, and a 1/Exp (B) suggesting a large odds ratio. Model 2 analyzed the effects of negative life events, CRHR1 methylation, Lh lateral orbitofrontal cortical thickness, and the interaction of these variables. Interaction of CRHR1 methylation and Lh lateral orbitofrontal thickness revealed a B of −0.43 which was marginally significant though the Exp (B) of 0.647 indicates a substantial protective effect (1 minus 0.647 = 0.353) or 35.3% reduction in SUD outcome.

In Model 3, the effects of methylation of POMC, negative life events near DNA collection, and the thickness of the parsopercularis in the right hemisphere were analyzed along with sex. The inclusion of sex in Model 3 was indicated by the interaction of familial risk and sex (Table 6). Model 3 results show a significant effect of POMC, LISRES negative life events, the thickness of the parsopercularis (Rh), and sex. Importantly, a significant interaction of these variables was seen (Table 7).

## 4. Discussion

This study was designed to evaluate the effects of a family history of alcohol use disorder on the cortical thickness of specific brain regions and relate these changes to event-related potentials recorded at the time of first evaluation in childhood at an average age of 11 years and at the time point closest to an MRI scan (an average of 24 years). With the familial risk for AUD potentially conferring greater risk for childhood adversity, negative life events assessed in childhood provided a window on the potential impact of childhood adversity on cortical thickness. The collection of DNA samples in childhood provided the opportunity to assess the epigenetic effects of childhood adversity. Two genes, CRHR1 and POMC, involved in the hypothalamic–pituitary–adrenal axis response to stress were studied using methylation assays. 

Previous work has shown a relationship between cortical thickness and family history of AUD in adolescents with those with a family history showing reduced thickness relative to those without a family history [53]. Evaluating the significance of variation in cortical thickness is complicated by developmental changes in cortical thickness [54]. Event-related potentials and related event-related oscillations have been studied extensively with the goal of providing potential biomarkers of familial risk. Developmental trajectories of specific ERP components have been demonstrated, showing age-related changes in amplitude and latency that are also related to familial risk for AUD [26]. Only a few studies have investigated the relationship between event-related potentials or event-related oscillations and cortical thickness. One study that assessed cortical thickness and P300 (P3a and P3b) found a relationship between the cortical thickness of the temporoparietal region and amplitude of P3a, and cortical thickness of the orbitofrontal cortex and P3b latency in a sample of 72 individuals ranging in age from 20 to 88 years [62].

In the present study, we found multiple significant correlations between P3 amplitude at Pz and cortical thickness with six of these differing by familial risk group. With the goal of determining how familial risk for AUD might change the relationship between ERP components and cortical thickness, we focused our analyses on those significant correlations that reached a *p* value of 0.01 or greater and showed patterns that differed in high and low-risk offspring. Interestingly, several significant correlations were seen in one familial risk group and not the other using ERP measures assessed at a point near the time of the scan. Using the most salient predictors from this analysis, Cox regression analyses were performed using SUD outcome as a measure of the strength of these predictors in influencing the development of SUD. Most of the ERP measures showing significance were those collected closest to the scan, though P300 latency recorded at the first ERP evaluation at a mean age of 11 years was a significant predictor. Cortical thickness of the parsopercularis in the right hemisphere was influential in the SUD outcome. Although the *p* value was marginal (0.096), the Exp (B) of 0.280 suggests that a substantial amount of variance was explained by parsopercularis (BA44) thickness. This finding is especially intriguing in view of the characteristics previously assigned to this region. The paropercularis has previously been identified as an area with mirror neurons (80), neurons thought to enable primates to imitate the actions of others and in motor learning of new skills [80]. Mirror neurons have also been discussed in the context of providing empathy for others [81]. One function of the parsopercularis may be to provide the substrate for motor-inhibitory performance in childhood and adolescence [82]. A comparison of cortical thickness of the parsopercularis in high- and low-risk participants found the high-risk participants have thinner cortices. 

Cortical thickness of the Lh lateral orbitofrontal region also emerged as a region predicting SUD outcome. This region has been characterized as having a substantial role in anticipating choices and integrating prior with current information [83,84]. The cortical thickness of the Lh lateral orbitofrontal region was significantly thinner in high-risk individuals than in their low-risk counterparts. 

We reasoned that these differences might be due to genetic or epigenetic variation experienced by those with differing familial backgrounds. Using longitudinal data for individuals with ERP recordings and MRI scans who had participated in repeated assessments in childhood gave us the opportunity to evaluate the impact of negative life events on epigenetic changes in two genes that play prominent roles in the stress response, the POMC and CRHR1 genes. Attenuated and enhanced HPA axis response to stress is maladaptive because of the dysregulated production of cortisol which can have negative consequences for both physical [85] and mental health [86]. We found significantly greater methylation changes in the CRHR1 gene that suggest reduced expression of this gene. One study of childhood maltreatment found reduced methylation of CRHR1, though no changes in gene expression were reported [87]. Reduced methylation has been reported in patients with panic disorder and in healthy controls with elevated Beck Depression Inventory scores [88]. 

Genetic variation in the CRHR1 gene has been related to phenotypic differences [89,90,91,92]. These studies have focused largely on three SNPs (rs110402, rs7209436, rs242924). The cortisol response to acute psychosocial stress, the Trier Social Stress Test, has been reported to be nominally associated with allelic variation in all three CRHR1 SNPs with interactions between trait anxiety and response to stress seen in association with rs110402 variation. The major allele of rs110402, a G nucleotide, has been reported to be associated with blunted cortisol reactivity in adolescents [90]. Variation in the G allele of rs110402 has also been reported to influence the functional magnetic imaging (fMRI) signal in the ventrolateral prefrontal cortex in response to negative emotional word processing [91]. The G allele of rs110402 has also been associated with fatigue and depression with the minor allele carrier having less of each [92]. A relationship between allelic variation and methylation has been shown in a study investigating the treatment effects of GSK561579, a CRF1 receptor antagonist, for women with post-traumatic stress disorder [93]. While the medication was not effective overall, it did show greater symptom reduction in those with CRF hyperactivity, namely those with a history of child abuse or those with a GG genotype. Significant differences in methylation were observed in those administered GSK561579 who were in the high CRF activity group suggesting that allelic variation influenced methylation. This phenomenon of allele-specific methylation has previously been described for many genes, but here points to both genetic and epigenetic modification effects. 

A familial risk group difference in methylation of the POMC gene was not found. However, when data were analyzed by sex, males were found to differ by familial risk while females were not. Our findings indicating hypermethylation of the promoter region within the POMC gene are especially interesting in view of previous reports that prenatal use of alcohol increases the stress response of exposed offspring in part due to the lowering of POMC expression through an epigenetic mechanism [94]. In the most recent work by these investigators [95] fetal alcohol-exposed rat offspring showed increased POMC methylation and reduced gene expression that was associated with increased plasma corticosterone response to restraint stress and increased anxiety-like behavior. 

In view of the POMC and CRHR1 methylation differences we observed along with differences in cortical thickness of the Lh lateral orbital and Rh parsopercularis regions, and greater negative life events seen in participants from high familial risk, it was of interest to determine if these variables would influence the likelihood and onset of SUD. Three models were tested with all showing significant Cox regression results. Most notable was the relationship between POMC with LISRES negative life events, sex, and cortical thickness of the Rh parsopercularis. 

Longitudinal follow-up of offspring at ultra-high risk for AUD or SUD through their membership in multiplex families has revealed an increase in both externalizing and internalizing disorders in comparison to low-risk controls {66]. These observations along with greater negative life events being reported by these high-risk children in comparison to controls confirm that early life adversity is more prominent in offspring from families in which multiple cases of alcohol use disorders are present in the parental generation. These experiences can be expected to profoundly affect the HPA stress response. Previous observations that childhood adversity is associated with brain morphological changes [46,47,48,49,50,51] are consistent with the present findings showing reduced cortical thickness in two regions, LH lateral orbitofrontal and RH parsopercularis. The present observations concerning CRHR1 methylation changes suggest a possible mechanism whereby chronic stress may lead to changes in methylation and gene expression that in turn affect cortical development. Although the results for POMC were not significant overall, the findings for males suggest that an additional gene involved in the HPA axis stress response may influence brain morphology. Finally, it is useful to reflect on electrophysiological differences that may be observed between high and low risk for AUD offspring from the perspective of early childhood adversity. While there have been multiple reports showing a relationship between specific genetic variants and the commonly studied P300 component of the ERP wave, epigenetic mechanisms, particularly those involving the HPA axis may explain some of the variation seen between risk groups.

## 5. Conclusions, Limitations, and Future Directions

Conclusions: In conclusion, the extensive literature now suggests that differences in event-related potentials and event-related oscillations seen in association with familial risk for AUD are salient endophenotypes of risk that have clarified the role of specific genes in this process. However, few studies have considered the role of the family environment in measuring electrophysiological characteristics. In contrast, the literature on early life adversity and brain morphology suggest that environmental factors play an important role in brain development. We suggest that the relationship between brain morphology and ERP characteristics uncovered by our longitudinal data collection across childhood, adolescence, and into young adulthood point to environmental adversity playing an important role in both. Because the hypothalamic–pituitary axis is key in the stress response to adversity, the epigenetic findings for CRHR1 and POMC are especially noteworthy. 

Limitations: The assessments of childhood adversity were based on self-report measures only. Reports from collateral reporters or home environment observations would be useful in further documenting the type and amount of early life adversity. However, one strength of the study design was the longitudinal data collection that enabled analysis to focus on environmental measurement closest to epigenetic measures with confirming evidence restricted just to cases where environmental assessment occurred before sample collection for epigenetic analysis.

Future Directions: The present results suggest the importance of measuring neurobiological consequences of adversity using a multimodal approach. Importantly, this approach should be incorporated into intervention trials designed to reduce adversity in families with AUD and SUD, particularly in children from families with multigenerational AUD characterized by multiple affected cases where the recurrence risk is so high. 

## Figures and Tables

**Table 1:** Demographic Characteristics of High-Risk and Low-Risk Adolescents and Young Adults (N=217).

	High-Risk (N=118) 54 Male and 64 Female		Low-Risk (N=99) 59 Males and 40 Females				
	Mean	SD	Mean	SD	T	df	p
Age at MRI Scan	26.18	4.47	24.13	5.15	3.15	1	0.002
Age at Nearest ERP	25.17	4.52	23.22	5.35	2.90	1/214	0.005
Age at First ERP	11.29	2.71	11.15	2.43	0.37	1/197	NS
Age at Last Follow up	26.90	4.68	24.98	5.06	2.91	1/215	0.004
BMI (at scan age)	27.89	6.27	27.41	6.15	0.56	1	NS
SES	39.92	10.55	45.70	8.87	4.32	1/215	0.019
PPVT (@118 ± 2.6 Years)	105.57	15.79	112.28	16.95	2.86	1/194	0.005
Number (%) Right-Handed	113 (95.8%)		90 (91%)		X^2^ = 2.10	1/215	NS
Alcohol or Drug Abuse/Dependence (Lifetime)	67		17		X^2^ = 35.60	1	<0.0001
Alcohol or Drug Abuse/Dependence < Scan	64		15		X^2^ = 35.52	1	<0.0001

**Table 2:** Bivariate correlations between amplitude and latency of ERP components and FreeSurfer regions in the left hemisphere (N=217).

Left Hemisphere	N1 Cz	N2 Cz	P2 Cz	P3 Pz	N1 Latency	N1 Latency	N2 Latency	P2 Latency	P3 Latency
Frontopole				0.008					
Lateral occipital			0.008						
Medial orbitofrontal									0.008
Lateral orbitofrontal				0.002					
Precuneous	0.002								
Rostral Mid Frontal				0.005					
Sup Frontal				0.009					
Sup parietal	0.004								
Temporalpole			0.003						

All FreeSurfer regions were correlated with all ERP components. Results are shown for correlations of<0.01. Only those that differed by risk are shown.

**Table 3:** Bivariate correlations between amplitude and latency of ERP components and FreeSurfer regions in the right hemisphere (N=217).

Right Hemisphere	N1 Cz	N2 Cz	P2 Cz	P3 Pz	N1 Latency	N1 Latency	N2 Latency	P2 Latency	P3 Latency
Inferior Parietal									0.004
Isthmus Cingulate	0.006								
Parsopercularis				<0.0001					
Postcentral			0.009						0.004
Sup Frontal				0.009					

All FreeSurfer regions were correlated with all ERP components. Results are shown for correlations of<0.01. Only those that differed by familial risk are shown.

**Table 4:** FreeSurfer regions associated with ERP components recorded at a point closest to the MRI scan ^a^.

		High Risk		Low risk	
Variable 1	Variable 2	R	P Value	R	P Value
Lh Frontopole	P3 amp@Pz	0.037	NS	0.329	0.001
Lh Lateral Occipital	P2 amp @ Cz	0.199	0.032	0.159	NS
Lh Lateraloribitofrontal	P3 amp@Pz	0.275	0.003	0.385	0.001
Lh Precuneous ^b^	N1 amp @ Cz	0.000	NS	-0.373	<0.001
Lh Rostral Midfrontal	P3 amp @Pz	0.045	NS	0.318	0.001
Lh Superior Frontal	P3 amp @Pz	0.102	NS	0.243	0.015
Lh Superior Parietal ^C^	N1 amp @ Cz	-0.062	NS	-0.311	0.002
Lh Medial Orbital	P3 latency	0.149	NS	0.185	0.067
Lh Tempotalpole ^d^	P2 amp @ Cz	0.109	NS	0.320	0.001
Rh Inferior Parietal	P3 latency	0.207	0.025	0.154	NS
Rh Isthmus Cingulate	N1 amp @ Cz	-0.182	0.049	-0.171	NS
Rh Parsopercularis	P3 amp @ Pz	0.179	0.053	0.304	0.002
Rh Postcentral ^e^	P2 amp @ Cz	0.218	0.018	0.129	NS
Rh Postcentral ^f^	P3 latency	0.162	0.080	0.230	0.022
Rh superior Frontal	P3 amp@Pz	0.129	NS	0.225	0.025

Correlations between variables were performed separately within the high and low risk groups. ^a^ Those differing by familial risk are shown. Lh lateralorbitofronal and rh. parsopercularis revealed significant correlations within each familial risk group and are shown due to their impact on SUD survival. ^b^ Lh precuneous was also significant for N1 amplitude recorded at the earliest age for low risk participants (r = −0.279, *p* = 0.009). ^c^ Lh superior parietal was also significant for N1 amplitude recorded at the earliest age for low risk participants (r = −0.224, *p* = 0.37). ^d^ Lh temporalpole was also significant for P2 amplitude recorded at the earliest age for low risk participants (r = 0.254, *p* = 0.018). ^e^ Rh postcentral was also significant for P2 amplitude recorded at the earliest age for high risk participants (r = 0.215, *p* = 0.023). ^f^ Rh postcentral was also significant for P3 latency recorded at the earliest age for high risk participants (r = 0.215, *p* = 0.023).

**Table 5:** Cox Regression-SUD Survival (N = 217) using selected ERP measures and selected brain regions.

	B	SE	Wald	df	p	Exp(B)
RH Parsopercularis Thickness	-1.275	0.766	2.76	1	0.096	0.280
N2_Cz_Closest to Scan	0.077	0.024	9.95	1	0.002	1.080
P2_Cz_Closest to Scan	-0.060	0.027	5.15	1	0.023	0.941
N2 Latency Closest to Scan	0.008	0.003	6.16	1	0.013	1.008
N1 Cz Earliest	-0.091	0.032	8.03	1	0.005	0.913
N2 Latency Earliest	0.007	0.003	5.59	1	0.018	1.007
P3 Latency Earliest	-0.003	0.002	3.92	1	0.048	0.997

Omnibus Test of Model Coefficients Chi Square = 31.26, df = 7, *p* < 0.0001.

**Table 6:** Risk group differences in FreeSurfer regions, CRHR1 methylation, Negative Life Events (NLE) scores closest to sample collection (before or after), and NLE scores restricted to just those obtained before sample collection.

	High Risk			Low-Risk			t	df	*p* Value
	N	MEAN	SD	N	MEAN	SD			
Lh lateral orbitofrontal	118	2.63	0.16	99	2.69	0.17	-2.74	215	0.007
Rh parsopercularis	118	2.54	0.16	99	2.60	0.16	-2.64	216	0.009
CRHR1 Methylation ^a^	105	0.93	0.17	71	0.86	0.21	2.32	126.4	0.022
POMC	99	0.273	0.05	71	-0.263	0.05	1.25	168	NS
POMC Males	47	0.280	0.03	45	0.254	0.06	2.75	90	0.007
POMC Females	52	0.265	0.05	26	0.278	0.04	-1.11	76	NS
NLE Close to Sample Collection ^b^	106	50.4	10.6	72	44.7	7.5	4.17	175.8	<0.001
NLE Before Sample Collection ^c^	42	54.6	11.4	47	46.8	7.8	3.7	71.9	<0.001

^a^ Percent methylation was transformed to logo (percent methylation +1). ^b^ NLE scores closest to the blood sample collection was chosen for analysis f NLE score was obtained either before or after sample collection (N=176). Mean interval between NLE assessment and blood sample collection was 1 65 ± 2.3 years. ^c^ NLE score obtained closest to blood sample collection but only if the score was obtained before the blood sample collection (N=87). Mean interval betwe en these variables was 0.01 ± 0.11 years.

**Table 7:** Cox Regression-SUD Survival (N = 217) with CHRH1 methylation, POMC methylation, Negative Life Events, parsopercularis, and lateralorbital cortical thickness.

	B	SE	Wald	df	p	Exp(B)
Model 1						
CRHR1 Methylation	19.682	10.983	3.21	1	0.073	3.53 × 10^8^
LISRES NLE Closest to DNA Collection	0.54	0.011	23.46	1	<0.001	1.06
Rh Parsopercularis	6.77	3.84	3.10	1	0.078	8.69 × 10^2^
CRHR1 Methylation ∗ RhParsopercularis	-8.31	4.34	3.66	1	0.056	<0.001
Model 2						
LISRES NLE Closest to DNA Collection	0.053	0.011	24.24	1	<0.001	1.05
CRHR1 Methylation ∗ Lh Lateral orbitofrontal	-0.43	0.257	2.86	1	0.091	0.647
Model 3						
POMC METHYLATION	26.89	10.38	6.71	1	0.01	4.78 × 10^11^
LISRES NLE Closest to DNA Collection	9/51	0.011	20.66	1	<0.001	1.05
Rh Parsopercularis	2.28	1.16	3.84	1	0.05	9.78
SEX	5.05	1.65	9.36	1	0.002	155.59
POMC ∗ LISRES NLE Closest to DNA Collextion ∗ SEX ∗ Rh Parsopercularis	-7.73	2.42	10.23	1	<0.001	<0.001

Omnibus Test of Model Coefficients Chi Square = 31.59, df = 4, *p* < 0.0001 for Model 1; Chi Square = 27.62, df = 2, *p* < 0.0001; for Model 2; 33.86, df = 5, *p* < 0.0001.

## Data Availability

No publicly available data sets are available because consent to share was not obtained for participants whose data was collected before the data sharing era.
